# Linking Cell Size, *V_max_* and *K_m_* in Phototrophs and Chemotrophs: Insights From Bayesian Inference

**DOI:** 10.1111/1758-2229.70114

**Published:** 2025-06-19

**Authors:** Risa Sasaki, Mayumi Seto

**Affiliations:** ^1^ Department of Chemistry, Biology, and Environmental Sciences Nara Women's University Kita‐Uoya Nishimachi Nara Japan

**Keywords:** cell size, chemotrophic organisms, mechanistic limitation, Michaelis–Menten model, Monod model, parameterisation, resource uptake rate, *V*_*max*_‐*K*_*m*_ tradeoff

## Abstract

Microbial growth is often described in terms of resource uptake rates, making the understanding and parameterisation of these rate‐limiting processes critical for microbial modelling. In phototrophic plankton, theoretical studies suggest that nutrient uptake is limited by mechanistic processes involving membrane transporters, and it has been observed that the cell‐specific maximum resource uptake rate (*V*_*max*_) follows a power‐law relationship with cell size, as well as a trade‐off between *V*_*max*_ and the half‐saturation constant (*K*_*m*_). These constraints may also apply to chemotrophic microorganisms; however, many datasets lack direct cell‐size measurements. We therefore leveraged the assumption that prokaryotic cell sizes, *V*_*max*_, and *K* _*m*_ each follow log‐normal distributions, drawing parallels with established phytoplankton scaling laws. Our analysis suggests that chemotrophic organisms generally exhibit higher maximum uptake rate per dry weight (*V*_*maxDW*_) and *K* _*m*_ values than phototrophs, and that *V*_*maxDW*_ and *K* _*m*_ are not strongly correlated when all chemotroph data are combined. Furthermore, the Bayesian‐derived exponents for *V*_*maxDW*_ and *K* _*m*_ exceeded those expected from allometric scaling relationships based on the membrane‐transport capacity observed for phototrophs, implying that a range of additional factors likely affect observed kinetic parameters.

## Graphical abstract

Using scaling laws and Bayesian estimation, we inferred the relationship between cell size and uptake kinetics in chemotrophs, finding higher scaling exponents than in phytoplankton, suggesting additional factors influence microbial resource uptake.

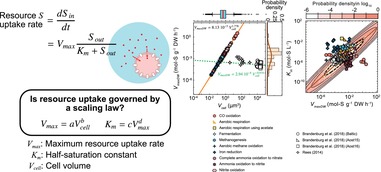

## 1 Introduction

Microbial metabolism and growth are critical drivers of biogeochemical processes, making it essential to understand and predict microbial population dynamics in order to forecast ecosystem services and environmental changes (Prosser et al. 2007; Widder et al. 2016). Regardless of resource type and internal metabolic pathway complexity, microbial growth rate is often described as proportional to the uptake rate of the most limiting resource (Butler and Wolkowicz 1985; Button 1985), suggesting that the uptake process serves as a bottleneck for both metabolic activity and growth kinetics. This notion is further supported by approaches such as flux balance analysis, which assumes that the intracellular metabolite concentrations reach a quasi‐steady state by rapidly adjusting to changes in external substrate concentrations, thereby enabling the estimation of cellular metabolite fluxes (Covert et al. 2001; Varma and Palsson 1994). Hence, obtaining accurate estimates of uptake kinetics is crucial for predicting microbial growth.

**FIGURE 1:**
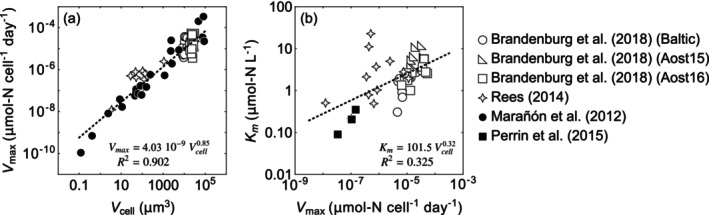
Scaling relationships based on available phytoplankton data. (a) The relationship between maximum cell‐specific uptake rates *V*_*max*_ and cell volume *V*_*cell*_. (b) The relationship between half‐saturation constants *K*_*m*_ and *V*_*cell*_. The dotted lines represent the regression equations. Data from Brandenburg et al. (2018): ‘Baltic’, ‘Aost15’ and ‘Aost16’ refer to strains isolated from the Baltic Sea in 2015 (Baltic), and from the Netherlands in 2015 (Aost15) and 2016 (Aost16), respectively.

The microbial resource uptake rate is often represented by the Monod equation or Michaelis–Menten equation (Bae and Rittmann 1996; Dugdale 1967; Monod 1949):

(1) $$\text{resource uptake rate}=V_{\max}\frac{S}{K_{m}+S}$$

where *S* (mol L^−1^) is the limiting resource concentration, *V*_*max*_ (mol S cell^−1^ h^−1^) is the maximum uptake rate, and *K*_*m*_ (mol S L^−1^) is the half‐saturation constant. Aksnes and Egge (1991) mechanistically derived both *V*_*max*_ and *K*_*m*_ based on cellular morphology and physical transport processes. Under the assumption that nutrient uptake in phytoplankton is limited by transporter uptake steps and that the cell is spherical, they expressed:

(2) $$V_{\max}=\left(h^{-1}n_{0}4\pi \right)r^{2}$$

(3) $$K_{m}={\left(\mathrm{AhD}\right)}^{-1}r$$

where *h* denotes the handling time per ion by a transporter, *n*_0_ represents the transporter site density, *r* is the radius of a cell, *A* is the area of one uptake site and *D* represents molecular diffusion. From Equation (2), if the transporter site density is constant, *V*_*max*_ is expected to scale with the 2/3 power of the cell volume (*V*_*cell*_), because cell volume scales as *V*_*cell*_ ∝ *r* ^3^, thus yielding *V*_*max*_ ∝ *r* ^2^ ∝ $V_{\text{cell}}^{\frac{2}{3}}$. Furthermore, *K*_*m*_ is predicted to scale with the square root of *V*_*max*_. Litchman et al. (2007) tested these predictions by compiling data on *V*_*cell*_ (μm^3^), *V*_*max*_ and *K*_*m*_ for phytoplankton utilising nitrate, and examined the power‐law relationship:

(4) $$V_{\max}\;\left(\text{μmol}\;S\;{\text{cell}}^{-1}\;{\mathrm{day}}^{-1}\right)={{\mathrm{aV}}_{\text{cell}}}^{b}$$

(5) $$K_{m}\;\left(\text{μmol}\;S\;L^{-1}\right)={{\mathrm{cV}}_{\max}}^{d}$$

with scaling exponents *b* = 0.67 and *d* = 0.357, respectively. The positive value of *d* indicates a trade‐off constraint, implying that organisms with higher *V*_*max*_ cannot achieve lower *K*_*m*_. While the underlying mechanism remains unclear, this pattern has been consistently observed not only across a wide range of phytoplankton species but also in chemotrophic organisms such as *Escherichia coli* (Brandenburg et al. 2018; Fiksen et al. 2013; Meyer et al. 2015; Smith et al. 2014, and see Figure 1 for visualisation of scaling relationships based on available phytoplankton data). However, it remains unclear whether this relationship holds universally across other chemotrophic organisms, whose growth can be limited by various electron donors and acceptors that serve as energy sources (Hoehler 2004; Hoehler and Jørgensen 2013; Lever et al. 2015).

The objective of this study is to examine the relationships among cell size, *V*_*max*_, and *K*_*m*_ in chemotrophic microorganisms based on published kinetic parameter data. However, a major obstacle is that direct comparisons between cell size and kinetic parameters are challenging because many available datasets reporting *V*_*max*_ and *K*_*m*_ in chemotrophs lack cell‐size information. To overcome this issue, we took an indirect approach: (i) given that prokaryotic cell sizes follow a lognormal distribution, we assumed that *V*_*max*_ and *K*_*m*_ would also follow lognormal distributions if Equations (4) and (5) hold; (ii) under these assumptions, we estimated the scaling parameters (*a*, *b*, *c* and *d*) using Bayesian inference on compiled data. This approach enabled us to infer the cell‐kinetic parameter relationships in chemotrophic microorganisms and compare them with those known from phytoplankton.

## 2 Materials and Methods

### 2.1 *V_max_* and *K*_*m*_ Reported in the Literature

We collected 161 reported values of *V*_*max*_ and *K*_*m*_ for chemotrophic organisms from 68 references (see Data Availability Statement), including some references that provided only *K*_*m*_ values. The chemotrophic organisms included both autotrophs and heterotrophs from bacteria, archaea, and single‐celled eukaryotes (yeast). They were classified based on their energy source reactions as reported in the source literature, without accounting for phylogenetic differences (see Table S1). This classification represents a reaction‐based subdivision within the broader nutritional classification framework, emphasising energy‐yielding reactions as a basis for further categorisation.

Since many studies reported *V*_*max*_ for chemotrophs as the substrate uptake rate per unit of protein mass or dry cell weight, the units of *V*_*max*_ were standardised to mol S g^−1^ DW h^−1^ (DW: dry weight), where S denotes either the electron donor or acceptor required for microbial growth. Methods for unit conversion are detailed in the database. Outliers were identified and removed using the FindAnomalies function in Mathematica 12 prior to analysis.

### 2.2 Conversion From Cell‐Specific to Dry‐Weight‐Specific Maximum Uptake Rate

To align with the unit convention of *V*_*max*_ in our database (mol S g^−1^ DW h^−1^), we applied the empirical relationship between cellular weight and volume, derived by Lynch et al. (2022) based on 68 diverse taxa samples:

(6) $$w\;\left(g\;{\text{cell}}^{-1}\right)=\alpha V_{\text{cell}}^{\beta }$$

where *α* = 10^−12.244^ and *β* = 0.920. Let *V*_*maxDW*_ represent the dry‐weight specific maximum uptake rate. Using Equations (4) and (5), the following relationships exist between *V*_*maxDW*_, *K*_*m*_ and *V*_*cell*_:

(7) $$V_{\text{maxDW}}\;\left(\mathrm{mol}\;S\;g^{-1}\;\mathrm{DW}\;h^{-1}\right)=\frac{V_{\max}\;\left(\mathrm{mol}\;S\;{\text{cell}}^{-1}\;h^{-1}\right)}{w}=\frac{a}{\alpha }V_{\text{cell}}^{b-\beta }$$

(8) $$K_{m}\;\left(\mathrm{mol}\;S\;L^{-1}\right)=c{\left(wV_{\text{maxDW}}\right)}^{d}=cw^{d}V_{\text{maxDW}}^{d}=\bar{c}{\left(\frac{a}{\alpha }V_{\text{cell}}^{b-\beta }\right)}^{d}$$

where $\bar{c}$ = $cw^{d}$. Since *V*_*max*_ is expected to scale with the 2/3 power of *V*_*cell*_ when uptake rate is regulated by membrane transporters, *V*_*maxDW*_ is likewise expected to scale with 2/3 − *β*, resulting in approximately −0.25. Meanwhile, *K*_*m*_ is predicted to scale with the square root of *V*_*maxDW*_, just as it does in response to *V*_*max*_. These expected values are derived under the assumption that cells are spherical, and therefore may not strictly apply to microorganisms with highly diverse surface area‐to‐volume ratios. Nonetheless, they provide a useful reference point for evaluating general scaling trends across different taxa.

### 2.3 Probability Distribution of *V_max_* and *K*_*m*_

The probability distribution of *V*_*maxDW*_ and *K*_*m*_ was derived using the change of variables method. We first assumed that cell size follows a lognormal distribution. This assumption is based on numerous observations and theoretical studies, which, although not perfect, report that cell size tends to follow a lognormal‐like distribution (Craigie and Cavalier‐Smith 1982; Jakobsen and Carstensen 2011; Hosoda et al. 2011; Amir 2014). Moreover, this assumption simplifies the transformation process when deriving the distributions of *V*_*maxDW*_ and *K*_*m*_, as it requires fewer parameters to infer than other distributions. Let the cell volume *V*_*cell*_ follow a log‐normal distribution with mean *μ* and standard deviation *σ*, denoted as LN(*μ*, *σ* ^2^):

(9) $$V_{\text{cell}}∼\mathrm{LN}\left(\mu ,\sigma^{2}\right)$$

with *μ* and *σ* estimated from the database of 5380 prokaryotic cell volume sizes obtained from the database compiled by Secaira‐Morocho et al. (2024) (*μ* = −0.74 and *σ* = 1.49). From Equation (7), we have ln *V*_*maxDW*_ = ln *a* − ln *α* + (*b* − *β*) ln *V*_*cell*_. Thus, *V*_*maxDW*_ also follows a log‐normal distribution:

(10) $$V_{\text{maxDW}}∼\mathrm{LN}\left(\ln\;a-\ln\alpha +\left(b-\beta \right)\mu ,{\left(b-\beta \right)}^{2}\sigma^{2}\right)$$

with the probability density function (PDF) *f*(*V*_*maxDW*_). Taking the logarithm of Equation (8) gives ln *K*_*m*_ = $\ln\;\bar{c}+d\left\{\ln\;\frac{a}{\alpha }+\left(b-\beta \right)\ln\;V_{\text{cell}}\right\}$, so *K*_*m*_ also follows a log‐normal distribution:

(11) $$K_{m}∼\mathrm{LN}\left(\ln\;\bar{c}+d\left\{\ln\;\frac{a}{\alpha }+\left(b-\beta \right)\mu \right\},d^{2}{\left(b-\beta \right)}^{2}\sigma^{2}\right)$$

with the PDF *f*(*K*_*m*_).

To estimate the parameters *a*, *b*, $\bar{c}$ and *d* of the PDFs in Equations (10) and (11), which governs the dataset $D={\left\{\left(V_{\text{maxDW},i},K_{m,i}\right)\right\}}_{i=1}^{N}$, we applied the Metropolis‐Hastings variant of the Markov chain Monte Carlo (MCMC) method. The log‐likelihood function was defined using Equations (10) and (11):

(12) $$L\left(a,b,\bar{c},d\;|\;D\right)=\sum_{i=1}^{N}\ln\;f\left(\left.K_{m,i}\;\right|\;a,b,\bar{c},d\right)+\ln\;f\left(V_{\text{maxDW},i}\;|\;a,b\right)$$

The initial values for *a*, *b*, $\bar{c}$ and *d* were derived based on the relationships described in Equations (10) and (11), using the means and standard deviations of the logarithmic values of *V*_*maxDW*_ and *K*_*m*_ for chemotrophs. Proposal distributions for each parameter were normal distributions, with standard deviations at 5% of their respective initial values. We performed 100,000 MCMC iterations, discarded the first 20,000 iterations as burn‐in. Posterior means and standard deviations were adopted as final parameter estimates, and convergence was confirmed by visual inspection of the trace plots.

## 3 Results

### 3.1 Distributions of *V_max_* and *K*_*m*_ for Chemotrophs

**FIGURE 2:**
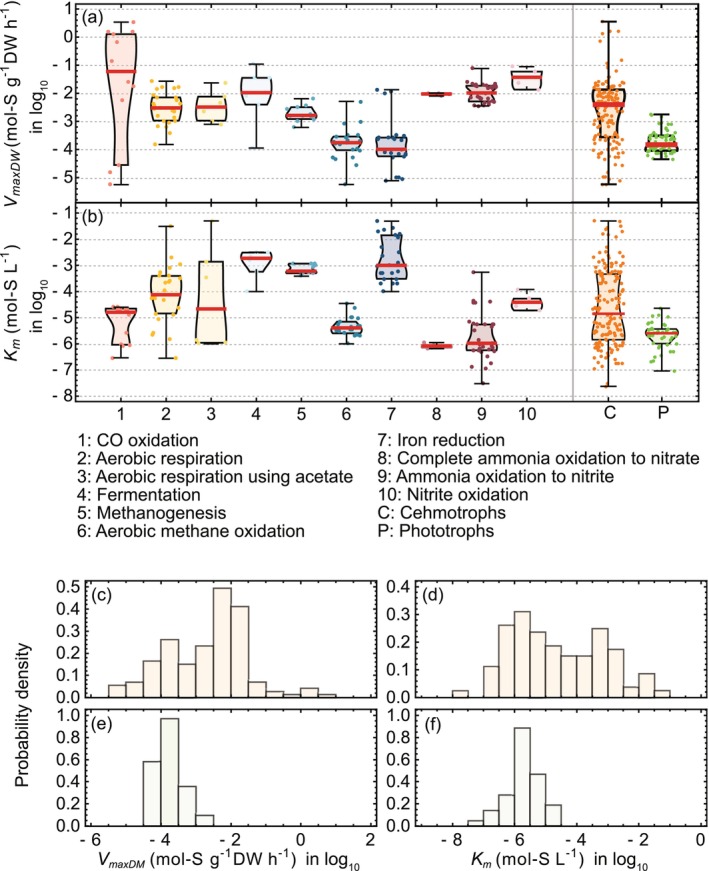
Distributions of maximum uptake rate per dry weight (*V*_*maxDW*_) and half‐saturation constants (*K*_*m*_) for chemotrophs reported in the literature. (a, b) Distributions of reported *V*_*maxDW*_ and *K*_*m*_ values for chemotrophs, categorised by their energy‐sourcing reactions. *V*_*maxDW*_ and *K*_*m*_ for chemotrophs represent the combined data of all categorised groups, while those for phototrophs correspond to the maximum uptake rate per dry weight, estimated using Equation (6) based on phototroph data with comparable cell sizes (Brandenburg et al. 2018; Rees 2014). (c, d) Probability density of *V*_*maxDW*_ and *K*_*m*_ for chemotrophs. (e, f) Probability density of *V*_*maxDW*_ and *K*_*m*_ for phototrophs.

The 161 reported values of *V*_*maxDW*_ and *K*_*m*_ for chemotrophs spanned several orders of magnitude (Figure 2). We compared these with *V*_*maxDW*_ and *K*_*m*_ for phototrophs; *V*_*maxDW*_ was estimated using Equation (6) based on phototroph data with comparable cell sizes. The median *V*_*maxDW*_ for chemotrophs was 3.82 mmol S g^−1^ DW h^−1^, whereas that for phototrophs was 0.14 mmol S g^−1^ DW h^−1^; similarly, the median *K*_*m*_ was 14 μmol S L^−1^ for chemotrophs, compared to 2.5 μmol S L^−1^ for phototrophs. In both cases, chemotrophs exhibited higher values, and Mann–Whitney tests confirmed these differences were statistically significant (*p* < 0.05).

Multiple normality tests (Shapiro–Wilk, Kolmogorov–Smirnov and Anderson–Darling tests) applied to the logarithmic values of *V*_*maxDW*_ and *K*_*m*_ for chemotrophs rejected the null hypothesis of normality. The assumption that *V*_*maxDW*_ and *K*_*m*_ follow a log‐normal distribution stems from the distribution of cell sizes, yet theoretical work suggests that cell sizes themselves are best characterised as ‘lognormal‐like’, rather than strictly log‐normal (Hosoda et al. 2011; Amir 2014). The 5380 prokaryotic cell volumes compiled by Secaira‐Morocho et al. (2024) likewise failed normality tests. Despite these results, we opted to treat cell volume, *V*_*maxDW*_, and *K*_*m*_ as log‐normally distributed in an approximate sense. We made this decision because *Q*–*Q* plots and histograms revealed that, while the tails deviate, the overall shape does not strongly depart from a log‐normal distribution (see Figure S2). Additionally, Hartigan's dip test in R supported the unimodality of both parameters (*p*‐values: 0.735 and 0.197, respectively), reinforcing that these parameters are at least single‐peaked. Nevertheless, as Kar et al. (2021) have pointed out, distributional assumptions such as log‐normality may introduce interpretational pitfalls if noise sources are not rigorously accounted for. In our study, we mitigate this risk by incorporating a Bayesian inference framework that explicitly captures variability in the data, thereby reducing the likelihood of drawing spurious conclusions based solely on an imperfect distributional assumption.

The observed variability in *V*_*maxDW*_ and *K*_*m*_ of chemotrophs may reflect differences in the physicochemical properties of the substrates they utilise. For example, although groups performing aerobic respiration commonly use oxygen and organic compounds, the specific organic substrates (e.g., acetate, glucose) vary widely in molecular size. Such differences may influence the handling time of substrates by transporters. Additionally, variations in the experimental system (e.g., temperature, pH) also contribute to the observed differences. Temperature, in particular, is known to affect metabolic activity by influencing various physiological and biochemical processes, including enzyme kinetics and the efficiency of transport proteins (Nedwell 1999; Gillooly et al. 2001; Price and Sowers 2004). However, the effects of temperature on uptake kinetics are highly complex, acting through multiple processes including the diffusion rate of substrates, which makes it challenging to apply a uniform temperature correction. This is further complicated by the fact that some data were obtained from thermophiles, whose metabolic responses to temperature may differ substantially from those of mesophiles.

In addition, observational errors contribute to variability, and some microbial groups may achieve disproportionately high uptake rates by bypassing transporter‐mediated uptake (Figure 2a,b). For *V*_*maxDW*_, bacteria utilising CO as an electron donor exhibited the highest values, exceeding 0.1 mol CO (g biomass)^−1^ h^−1^ (Figure 2a). Notably, CO uptake rate estimates are prone to errors due to CO's leakiness and high reactivity. Moreover, CO uptake is unlikely to be limited by transporter sites and is more likely governed by the diffusion process across the cell membrane (Möller et al. 2019). For *K*_*m*_, relatively higher values were observed in bacteria utilising Fe(III) as an electron acceptor. In such iron‐oxidising bacteria, instead of internalising iron oxides, these microbes adhere to surfaces and facilitate electron flow via extracellular electron shuttling (Glasser et al. 2017; Shi et al. 2016). This mechanism may explain why Fe(III)‐utilising kinetics deviate from transporter‐limited uptake mechanisms.

### 3.2 Relationship Between Reported *V*_*maxDW*_ and *K*_*m*_ for Chemotrophs

**FIGURE 3:**
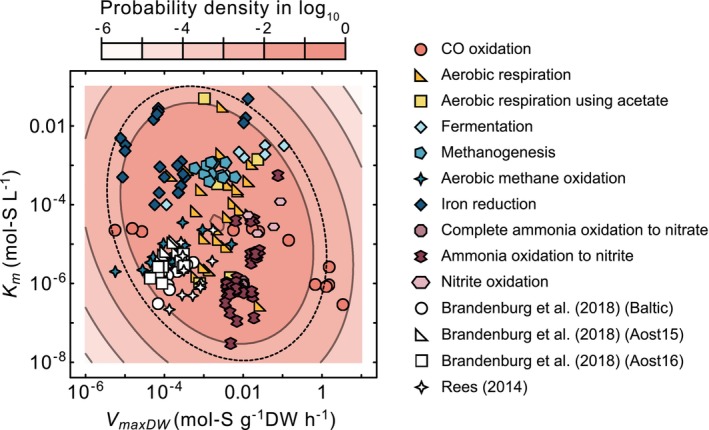
The relationship between *V*_*maxDW*_ and *K*_*m*_, along with the probability distribution fitted to the reported *V*_*maxDW*_ and *K*_*m*_ values for chemotrophs using a bivariate normal distribution. This distribution were obtained after applying a base‐10 logarithmic transformation to *V*_*maxDW*_ and *K*_*m*_. The mean and covariance of the fitted distribution are given as μ_1_ = −2.669, μ_2_ = −4.474, *σ*_11_ = 1.298, *σ*_12_ = −0.400 and *σ*_22_ = 2.025. The dotted circle represents the 95% confidence region, determined using the Mahalanobis distance corresponding to the 0.95 quantile of the Chi‐squared distribution with two degrees of freedom. Markers of different shapes in white correspond to the maximum uptake rate per dry weight, estimated using Equation (6) from phototroph data with comparable cell sizes.

After log‐transforming all *V*_*maxDW*_ and *K*_*m*_ values, the covariance was −0.40 and the adjusted *R* ^2^ value was 0.054. This indicates that, overall, there is a very weak linear association, suggesting that the two variables behave almost independently (Figure 3). In other words, for the *V*_*maxDW*_ and *K*_*m*_ dataset for chemotrophs, *K*_*m*_ does not follow a power‐law relationship with *V*_*maxDW*_, contrary to what is expected based on the relationship observed in phytoplankton (see Figure 1). When the same linear regression was conducted within each functional type with sample sizes *n* > 3, a correlation (*R* ^2^ > 0.2) was observed only for CO oxidation type (*R* ^2^ = 0.575), fermentation type (*R* ^2^ = 0.886), complete ammonia oxidation (COMAMMOX) type (*R* ^2^ = 0.402) and ammonia‐oxidising to nitrite type (*R* ^2^ = 0.26) (see Figure S2).

Among these, the CO oxidation type exhibited a negative exponent (*d* in Equation 5), indicating that *K*_*m*_ decreases with increasing *V*_*max*_, which contrasts with the trade‐off observed between *V*_*max*_ and *K*_*m*_ in phytoplankton species (see Section 4). For the fermentation type, the exponent was 0.529, close to the square root of *V*_*max*_. In contrast, the COMAMMOX type and ammonia‐oxidising to nitrite types exhibited larger but similar exponents. Both COMAMMOX and ammonia‐oxidising to nitrite types utilise NH_3_ and NH_4_^+^, suggesting that either the substrate used or ecological niche similarities may drive the comparable response of *K*_*m*_ to *V*_*max*_.

### 3.3 Bayesian Estimation of *V*_*maxDW*_ and *K*_*m*_ Distributions and Their Relationship to Cell Sizes

**FIGURE 4:**
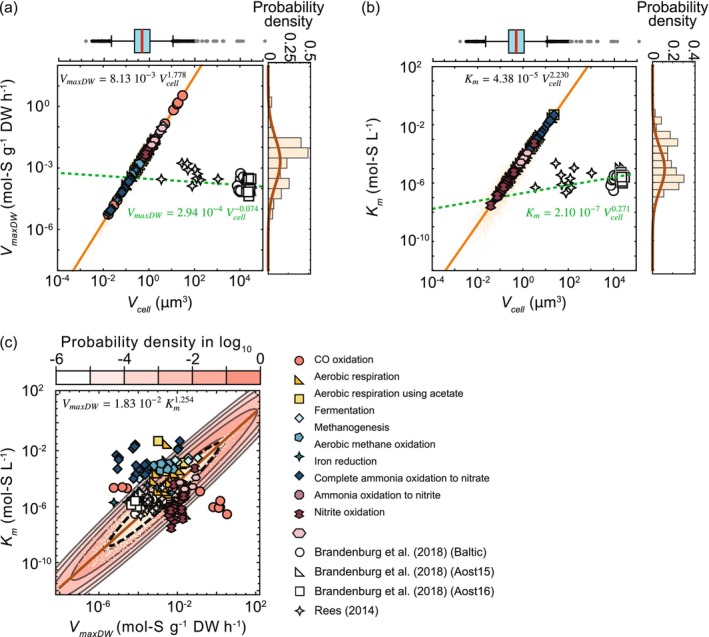
Estimated relationship among *V*_*cell*_, *V*_*maxDW*_, and *K*_*m*_. (a, b) Estimated relationships between *V*_*cell*_ and *V*_*maxDW*_ (a) and *K*_*m*_ (b). The orange line represents the relationship estimated using the posterior means of parameters *a*, *b*, $\bar{c}$ and *d* from Equations (10) and (11), based on chemotrophs' *V*_*maxDW*_ and *K*_*m*_. The orange dots indicate values of *V*_*cell*_, *V*_*maxDW*_, and *K*_*m*_, generated assuming normal posterior distributions for *a*, *b*, $\bar{c}$ and *d*, with *V*_*cell*_ following Equation (9). The green dashed line shows the relationship obtained from phytoplankton data in Figure 1 using Equation (7) with variable/unit conversions. The histograms on the right display chemotrophs' *V*_*maxDW*_ and *K*_*m*_ distributions, with overlaid probability distributions derived from posterior parameter means. The boxplot at the top represents prokaryotic cell size distributions from Secaira‐Morocho et al. (2024). (c) Estimated relationship between *V*_*maxDW*_ and *K*_*m*_ with a fitted probability distribution. The distribution was obtained by applying a base‐10 logarithmic transformation to the generated *V*_*maxDW*_ and *K*_*m*_. The mean and covariance of the fitted distribution are given as μ_1_ = −2.68, μ_2_ = −5.13, *σ*_11_ = 1.37, *σ*_12_ = 1.72 and *σ*_22_ = 2.28. The dotted circle represents the 95% confidence region, determined using the Mahalanobis distance for the 0.95 quantile of the chi‐squared distribution with two degrees of freedom.

Based on the *V*_*maxDW*_ and *K*_*m*_ values for chemotrophs, we obtained the scaling parameters *a*, *b*, $\bar{c}$ and *d* by Bayesian inference. The estimated means and standard deviations were *a* = (4.64 ± 1.14) × 10^−15^, *b* = 2.70 ± 0.11, $\bar{c}$ = (1.83 ± 0.64) × 10^−2^ and *d* = 1.254 ± 0.105 (see Figure S3). The log‐normal distribution obtained using these parameters successfully explained the distribution of *V*_*maxDW*_ and *K*_*m*_ for chemotrophs (the right‐side histograms in Figure 4a,b). The exponent parameter *b* significantly exceeds the 2/3 value predicted if *V*_*max*_ is mechanistically determined by cell size. As indicated by Equations (10) and (11), *b* influences the variance component of *V*_*maxDW*_. The relatively large value of *b* can be attributed to the model's need to accommodate substantial observational noise and data spread, resulting in increased dispersion. Similarly, the estimated *d* exceeds the 0.5 exponent predicted by the scaling relationship for *V*_*max*_.

By substituting these parameters into Equations (7) and (8), we estimated the relationships between *V*_*cell*_ and *V*_*maxDW*_, as well as *V*_*cell*_ and *K*_*m*_. The predicted range of cell sizes based on *V*_*maxDW*_ and *K*_*m*_ for chemotrophs was approximately 0.02–30 μm^3^. To contextualise these estimates, we compared them with the 5380 prokaryotic cell volumes compiled by Secaira‐Morocho et al. (2024), whose first and third quartile cell sizes were 0.218 and 1.048 μm^3^, respectively (see the top box‐and‐whisker plots in Figure 4a,b). Around 35% of the *V*_*cell*_ estimates based on *V*_*maxDW*_ and *K*_*m*_ fell within this range. To further evaluate the accuracy of these estimates, we compared them with reported cell size measurements of *Shewanella putrefaciens* , an iron reducer. Among the *V*_*maxDW*_ and *K*_*m*_ data we compiled, 23 data pairs were reported for *S. putrefaciens* . A separate study by the same author reported a dry weight per cell of 3 × 10^−13^ g dry cell^−1^ under similar experimental conditions (Bonneville et al. 2006). Applying this value to Equation (6) yielded an estimated cell volume of 0.5 μm^3^, whereas the cell volumes inferred from *V*_*maxDW*_ ranged from 1.6 to 17.5 μm^3^. This discrepancy suggests that, even if the assumed scaling relationships among cell volume and kinetic parameters hold, uncertainties in the estimated parameters can lead to predictions deviating by as much as 3.2–35 times. It is also worth noting that cell volume in batch culture can vary considerably depending on the growth phase or nutrient status (Roller et al. 2023). Consequently, relying on a single dry mass measurement as a universal reference introduces additional uncertainties when comparing *V*_*maxDW*_‐derived volumes.

Additionally, the estimated relationships between *V*_*cell*_ and *V*_*maxDW*_, as well as *V*_*cell*_ and *K*_*m*_, for chemotrophs differed markedly from those observed in phytoplankton. Intriguingly, a subset of phytoplankton data does overlap with the chemotrophic scaling relationship between *V*_*maxDW*_ and *K*_*m*_ (Figure 4c). However, interpreting these observations is challenging due to the large uncertainties in the Bayesian‐derived parameters and the limited number of direct comparisons available. Future studies that measure cell size, *V*_*max*_, and *K*_*m*_ under standardised temperature and pH conditions would be instrumental in clarifying whether these apparent differences (or similarities) reflect fundamental biological distinctions or simply arise from measurement constraints and variability.

## 4 Discussion

Trade‐offs, defined as negative correlations between two traits that enhance an organism's fitness, are known to constrain growth and distribution of organisms across all biological levels, including microorganisms (Ferenci 2016; Kneitel and Chase 2004; Werner and Anholt 1993). These constraints are often linked to physical and chemical factors, such as resource allocation (Kim et al. 2020; Weiße et al. 2015), and can be reinforced by selective pressures (Roff et al. 2002). The *V*_*max*_–*K*_*m*_ trade‐off has been reported to mechanistically arise from cell surface limitations for membrane transporters (Aksnes and Egge 1991), and this mechanism may broadly apply to unicellular organisms. However, our findings indicate that this *V*_*max*_–*K*_*m*_ trade‐off is not universally consistent, suggesting that these constraints may be modified or influenced by alternative physiological mechanisms.

Substrates like gaseous or solid species present unique challenges for microbial resource uptake. For microbes utilising such substrates without relying on transporters, the *V*_*max*_–*K*_*m*_ trade‐off may not apply. Alternatively, if an increase in uptake sites enhances both *V*_*max*_ and volume‐specific uptake affinity, or if resource handling time decreases independently of *V*_*max*_, the trade‐off can be mitigated (Fiksen et al. 2013). For example, ATP‐binding cassette transporters, widely used across kingdoms, may increase *K*_*m*_ independently of *V*_*max*_ by boosting the abundance of high‐affinity substrate‐binding proteins (Bosdriesz et al. 2015; Norris et al. 2021). However, this mechanism likely incurs an energetic cost, potentially leading to another trade‐off between nutrient uptake efficiency and growth rate (Ferenci 2016). In yeast, the presence of multiple transporters with varying affinities has been proposed as a mechanism to alleviate the *V*_*max*_–*K*_*m*_ trade‐off (Montaño‐Gutierrez et al. 2022).

In addition to physiological mechanisms, environmental factors may plastically influence *V*_*max*_ and *K*_*m*_. For instance, the *V*_*max*_ of fungal pathogens, yeast, and *E. coli* has been shown to decrease with increasing initial nutrient concentration during cultivation (Nev et al. 2021). Furthermore, the enzymatic reaction rate in steady state is theoretically optimised when *K*_*m*_ matches the substrate concentration (Ooka et al. 2023), which aligns with findings that *K*_*m*_ values for extracellular enzymes in diverse aquatic and terrestrial microbial communities approximately match ambient substrate concentrations (Sinsabaugh et al. 2014). Such evidence suggests that *V*_*max*_ and *K*_*m*_ for a given species are not fixed, but instead vary in response to environmental conditions and cultivation designs.

Our findings indicate that the traditional *V*_*max*_–*K*_*m*_ trade‐off may not apply uniformly across all microorganisms, particularly for those that utilise substrates without transporter‐mediated uptake. Instead, alternative physiological mechanisms and environmental plasticity likely play significant roles in shaping resource uptake dynamics in chemotrophs. These results underscore the importance of developing more nuanced models that incorporate both physical and environmental factors to better estimate microbial growth kinetics and advance the integration of theoretical and experimental geochemical research.

## 5 Conclusions

This study investigated the relationships among cell size, *V*_*max*_ and *K*_*m*_ in chemotrophic microorganisms by compiling literature data and employing a Bayesian inference approach. Because many datasets lacked direct cell‐size information, we treated prokaryotic cell sizes, *V*_*max*_, and *K*_*m*_ as log‐normally distributed, leveraging the scaling principles proposed in phytoplankton studies to indirectly estimate scaling parameters for chemotrophs. Our findings reveal that chemotrophic organisms generally exhibit higher *V*_*maxDW*_ and *K*_*m*_ values than phototrophs, and that there is no clear correlation between *V*_*maxDW*_ and *K*_*m*_ when all chemotroph data are combined. The Bayesian‐derived exponents for *V*_*maxDW*_ and *K*_*m*_ exceeded those anticipated from allometric scaling, which was originally linked to the relationship between cell size and membrane transporters, suggesting that factors beyond cell size (e.g., substrate type, physicochemical conditions, and measurement variability) likely influence observed kinetic parameters. Overall, these results underscore the complexity of linking kinetic parameters to cell size and highlight the need for more direct and standardised measurements of cell size, *V*_*max*_, and *K*_*m*_. Future work employing direct cell size measurements and consistent culture controls will help refine the scaling relationships and enhance our mechanistic understanding of microbial resource acquisition under diverse environmental and metabolic regimes.

## Author Contributions

**Risa Sasaki:** investigation, writing – original draft, visualization, validation, writing – review and editing, formal analysis, methodology. **Mayumi Seto:** conceptualization, methodology, writing – original draft, writing – review and editing, visualization, validation, funding acquisition, investigation, software, formal analysis, supervision, project administration, data curation.

## Conflicts of Interest

The authors declare no conflicts of interest.

## Supporting information


**Figure S1.** *Q*–*Q* plots of *V*_*maxDW*_ and *K*_*m*_ in chemotrophs.
**Figure S2.** Correlation and linear regression lines describing the relationship between the maximum uptake rate (*V*_*maxDW*_) and the half‐saturation constant (*K*_*m*_) for each functional group categorised by the energy‐sourcing reactions listed in Table S1.
**Figure S3.** Traces and posterior distributions of parameters *a*, *b*, $\bar{c}$ and *d* estimated using the Markov chain Monte Carlo method. The first 20,000 iterations were discarded as burn‐in.

**Supplementary Table S1.** Classification of chemotrophic organisms based on their energy‐sourcing reactions, along with the base‐10 log‐transformed mean (range: min to max) and standard deviation (SD).

## Data Availability

All code was written in the Wolfram Language platform using Mathematica 12. All code and data supporting the results have been archived in Dryad: https://doi.org/10.5061/dryad.s4mw6m9h2.
