# Supplementary material for: Linking Cell Size, Vmax and Km in Phototrophs and Chemotrophs: Insights From Bayesian Inference
Source: Environ Microbiol Rep. 2025 Jun 19;17(3):e70114. doi: 10.1111/1758-2229.70114 (PMC12178613; doi:10.1111/1758-2229.70114)
Supplement: Supplementary file 1 — Figure S1. Q–Q plots of V maxDW and K m in chemotrophs. Figure S2. Correlation and linear regression lines describing the relationship between the maximum uptake rate (V maxDW ) and the half‐saturation constant (K m ) for each functional group categorised by the energy‐sourcing reactions listed in Table S1. Figure S3. Traces and posterior distributions of parameters a, b, c¯ and d estimated using the Markov chain Monte Carlo method. The first 20,000 iterations were discarded as burn‐in. [file EMI4-17-e70114-s002.pdf]

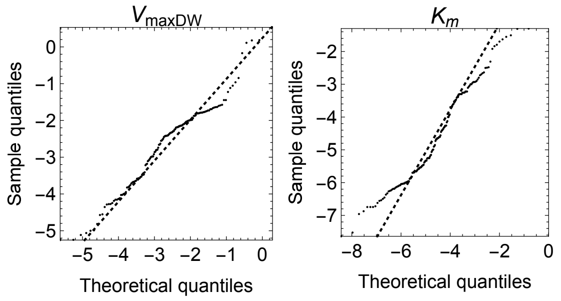

Figure S1: Q-Q plots of V_{maxDW} and K_m in chemotrophs.

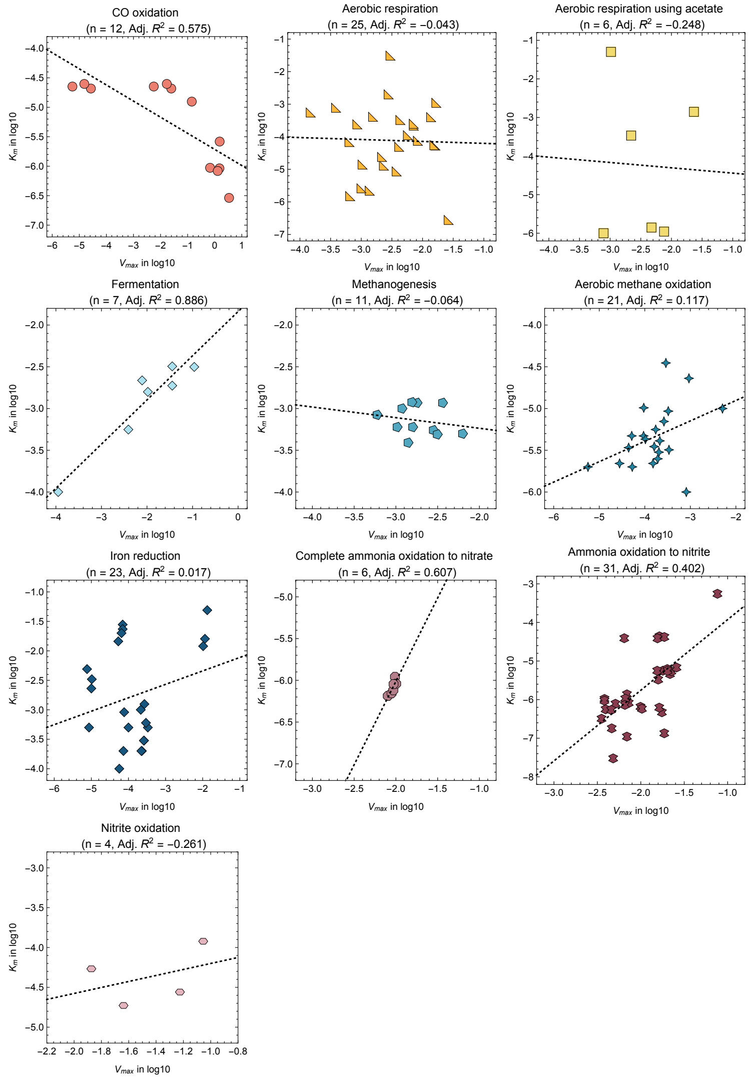

Figure S2: Correlation and linear regression lines describing the relationship between the maximum uptake rate (Vmax) and the half-saturation constant (Km) for each functional group categorized by the energy-sourcing reactions listed in Supplementary Table 1.

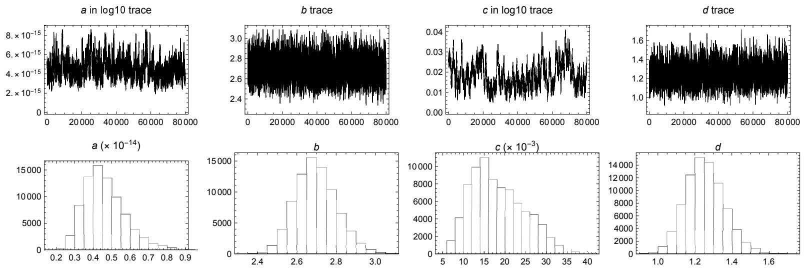

Figure S3: Traces and posterior distributions of parameters a, b, $\bar{c}$, and d estimated using the Markov chain Monte Carlo method. The first 20,000 iterations were discarded as burn-in.
