# Supplementary material for: Linking Cell Size, Vmax and Km in Phototrophs and Chemotrophs: Insights From Bayesian Inference
Source: Environ Microbiol Rep. 2025 Jun 19;17(3):e70114. doi: 10.1111/1758-2229.70114 (PMC12178613; doi:10.1111/1758-2229.70114)
Supplement: Supplementary file 2 — Supplementary Table S1. Classification of chemotrophic organisms based on their energy‐sourcing reactions, along with the base‐10 log‐transformed mean (range: min to max) and standard deviation (SD). [file EMI4-17-e70114-s001.docx]

**Supplementary Table 1**: Classification of chemotrophic organisms based on their energy-sourcing reactions, along with the base-10 log-transformed mean (range: min to max) and standard deviation (S.D.).

| Energy-sourcing reactions | Substrate used for the estimation of *V_maxDW_* and *K_m_* | log_10_*V_maxDW_* | | log_10_*K_m_* | | Sample size  (Outliers removed) |
| --- | --- | --- | --- | --- | --- | --- |
|  |  | Mean (min. to max.) | S.D. | Mean (min. to max.) | S.D. |  |
| 1. CO oxidation 2. Aerobic respiration 3. Aerobic respiration using acetate 4. Fermentation 5. Methanogenesis 6. Aerobic methane oxidation 7. Iron reduction 8. Complete ammonia oxidation to nitrate 9. Ammonia oxidation to nitrite 10. Nitrite oxidation | CO  Various carbohydrate excluding acetate  Acetate  Glucose  Acetate  Methane  Various iron oxides  Ammonia  Ammonia  Nitrite | −1.69 (− 5.25 to 0.53)  −2.53 (−3.83 to –1.57)  −2.47 (−3.11 to –1.63)  −2.04 (−3.95 to –0.96)  −2.72 (−3.22 to −2.20)  −3.79 (−5.25 to –2.29)  −3.82 (−5.12 to –1.88)  −2.04 (−2.10 to –2.00)  −1.98 (−2.45 to –1.12)  −1.45 (−1.87 to –1.05) | 2.12  0.57  0.56  0.97  0.29  0.60  0.91  0.04  0.32  0.37 | −5.25 (− 6.53 to – 4.60)  −4.11 (−6.56 to –1.51)  −4.24 (−6.00 to –1.30)  −2.92 (−4.00 to –2.50)  −3.14 (−3.41 to –2.92)  −5.34 (−6.00 to –4.45)  −2.76 (−4.00 to –1.31)  −6.08 (−6.19 to –5.96)  −5.71 (−7.52 to –3.26)  −4.37 (−4.73 to –3.92) | 0.74  1.12  1.99  0.54  0.18  0.37  0.83  0.08  0.91  0.35 | 14  25  6  7  11  21  23  6  37  4 |
